# Copy Number Variation of Age-Related Macular Degeneration Relevant Genes in the Korean Population

**DOI:** 10.1371/journal.pone.0031243

**Published:** 2012-02-15

**Authors:** Jung Hyun Park, Seungbok Lee, Hyeong Gon Yu, Jong-Il Kim, Jeong-Sun Seo

**Affiliations:** 1 Department of Ophthalmology, Seoul National University College of Medicine, Seoul, Korea; 2 Department of Ophthalmology, Seoul Paik Hospital, Inje University, Seoul, Korea; 3 Genomic Medicine Institute (GMI), Medical Research Center, Seoul National University, Seoul, Korea; 4 Department of Biomedical Sciences, Seoul National University Graduate School, Seoul, Korea; 5 Department of Biochemistry, Seoul National University College of Medicine, Seoul, Korea; 6 Psoma Therapeutics, Seoul, Korea; 7 Macrogen, Seoul, Korea; Radboud University Nijmegen Medical Centre, The Netherlands

## Abstract

**Purpose:**

Studies that analyzed single nucleotide polymorphisms (SNP) in various genes have shown that genetic factors are strongly associated with age-related macular degeneration (AMD) susceptibility. Copy number variation (CNV) may be an additional type of genetic variation that contributes to AMD pathogenesis. This study investigated CNV in 4 AMD-relevant genes in Korean AMD patients and control subjects.

**Methods:**

Four CNV candidate regions located in AMD-relevant genes (*VEGFA*, *ARMS2/HTRA1*, *CFH* and *VLDLR*), were selected based on the outcomes of our previous study which elucidated common CNVs in the Asian populations. Real-time PCR based TaqMan Copy Number Assays were performed on CNV candidates in 273 AMD patients and 257 control subjects.

**Results:**

The predicted copy number (PCN, 0, 1, 2 or 3+) of each region was called using the CopyCaller program. All candidate genes except *ARMS2/HTRA1* showed CNV in at least one individual, in which losses of *VEGFA* and *VLDLR* represent novel findings in the Asian population. When the frequencies of PCN were compared, only the gain in *VLDLR* showed significant differences between AMD patients and control subjects (*p* = 0.025). Comparisons of the raw copy values (RCV) revealed that 3 of 4 candidate genes showed significant differences (2.03 vs. 1.92 for *VEGFA*, *p*<0.01; 2.01 vs. 1.97 for *CFH*, p<0.01; 1.97 vs. 2.01, *p*<0.01 for *ARMS2/HTRA1*).

**Conclusion:**

CNVs located in AMD-relevant genes may be associated with AMD susceptibility. Further investigations encompassing larger patient cohorts are needed to elucidate the role of CNV in AMD pathogenesis.

## Introduction

Age-related macular degeneration (AMD) is a major cause of visual loss in the elderly in industrialized countries, estimated to affect more than 50 million people worldwide [1], [2]. Although the exact causes of AMD remain obscure, genetic factors are known to be responsible for AMD development, along with environmental and ocular factors [2], [3].

Genome-wide linkage and association studies have successfully identified several major chromosomal regions, including 1q31 (*CFH*) and 10q26 (*ARMS2/HTRA1*) [4], [5]. Along with *CFH* and *ARMS2/HTRA1*, genes associated with inflammation, oxidative stress, angiogenesis and lipid metabolism have been reported to be responsible for AMD susceptibility [6], [7], [8], [9].

We have previously discovered common Asian copy number variations (CNVs) through genotyping 30 individuals from 3 Asian populations - Korean, Chinese and Japanese. Using microarrays with 24 million probes, we have discovered 5,177 CNVs, of which 3,547 are putative Asian-specific CNVs [10]. A few studies have analyzed CNVs of AMD-relevant genes in Caucasians [11], [12]. However, the association of the Asian-specific CNVs of these genes with AMD has not been studied yet.

To verify if the CNVs of AMD-relevant genes are associated with AMD, we selected candidate CNVs discovered in the Asian populations and analyzed their status in Korean AMD patients and control subjects.

## Methods

### Study population

Consecutive, unrelated AMD patients and control subjects were recruited from the outpatient clinic at a research hospital from August 2008 to February 2010. The institutional review board of the Seoul National University Hospital approved this study, and written informed consents were obtained from all participants prior to study entry.

Color photographs of fundi were evaluated using the Age-Related Eye Disease Study (AREDS) grading system by graders blinded to patient diagnoses [13]. Patients were examined for the presence of drusen (including appearance and size), pigmentary abnormalities, geographic atrophy (GA), and choroidal neovascularization. Eyes with only a few small drusen were assigned Grade 1. Eyes with intermediate drusen were assigned Grade 2. Eyes with large confluent drusen or with pigmentary changes of the retinal pigment epithelium (RPE) were assigned Grade 3. Eyes with advanced changes, such as GA or choroidal neovascularization, were assigned Grade 4. Eyes with none of the above were assigned Grade 0. Patients were graded based on the worse eye. When choroidal neovascularization was suspected, fluorescein angiography was performed to confirm its presence. Subjects with Grade 0 were enrolled as controls. Because patients with a few small drusen have nominal AMD risk [13], subjects with Grade 1 were excluded from the AMD group. Only subjects older than 50 years were included in this study. In total, 273 AMD patients and 257 control subjects were enrolled. Information obtained included relevant past medical history, smoking history, and ocular history. Height, weight and body mass index (BMI) measurements were also recorded.

### DNA extraction and quantification

Ten milliliter (10 mL) of peripheral blood sample was collected from each study subject, and centrifuged for 5 min at 2000 g to separate the serum. DNA was extracted using FlexiGene DNA kit (Qiagen. Inc, Hilden, Germany) according to the manufacturer's protocol and stored at −70°C. DNA concentrations were measured by Nanodrop ND-1000 spectrophotometer (Nano-drop Technologies, Wilmington, DE). Samples from AMD patients and control subjects were collected during the same period and processed using the same method.

### Selection of target regions and copy number determination

Among CNVs discovered previously in the Asian populations, we selected candidate CNVs that overlapped with genes currently suspected to play crucial roles in AMD pathogenesis. The number of candidate CNVs was narrowed down to 4, each located in *VEGFA*, *ARMS2/HTRA1*, *CFH* and *VLDLR* (Tables 1 and S1).

**Table 1:** Target CNVs on causative genes.

Affected Gene	Mapped ID	CNV start	CNV end	Gain Suspected/Loss Suspected Among 30 Individuals
*VEGFA*	NM_001025369	43845963	43846991	1 (JPT 1)/0
*ARMS2/HTRA1*	NM_002775	124183770	124255215	1 (JPT 1)/0
*CFH*	NM_000186	194975094	195095411	0/5 (CHB 3, JPT 2*)
*VLDLR*	NM_003383	2610547	2613694	1 (CHB 1)/0

*CHB: Han Chinese from Beijing, JPT: Japanese from Tokyo.

We performed real-time PCR to determine CNV status on each sample. TaqMan probes, which were designed by the manufacturer (Applied Biosystems, Foster City, CA), were used to target the specific regions. *RNase P* (Taqman Copy Number Reference Assay, Applied Biosystems, Foster City, CA) was chosen as a reference gene, of which every human is known to possess 2 copies. Every reaction was duplicated in 384 well plates, and each plate was comprised of almost an equal number of disease and control samples. Real-time PCR was performed using the Applied Biosystems 7900HT Fast System and Sequence Detection Systems Software v2.3. The thermal cycling condition was as follows: holding at 95°C for 10 min, 40 cycles for 15 s at 95°C for denaturation and 60 s at 60°C for amplification.

Applied Biosystems CopyCaller™ Software v1.0 was used to determine the copy number status of each target region, and calculation was performed according to a maximum-likelihood algorithm of the software. Raw copy value (RCV) represents a non-integer number of copy calculated, whereas predicted copy number (PCN) is defined as an integer number of copy determined by the algorithm (0, 1, 2, or 3+). Copy number (CN) gain is defined as PCN higher than 2, and PCN lower than 2 would be regarded as CN loss.

### Statistical method for analysis

We performed a two-tailed t-test to evaluate the difference in mean values of RCV between the disease and control groups. A chi-square test was used to compare the frequency of CN gain and loss respectively between the two groups. For both analyses, statistical significance was deemed to have been reached when the *p*-value was lower than 0.05 (*p*<0.05).

## Results

The clinical characteristics of AMD patients and control subjects are summarized in Table 2. Among 273 AMD patients, neovascular AMD was diagnosed in 126 and non-neovascular AMD in 147 patients. Seventy-six patients had AMD of grade 2, 67 patients had AMD of grade 3 and 130 patients had AMD of grade 4, The mean age and gender distributions were not significantly different between the AMD and control groups. There was no significant difference in BMI between the two groups.

**Table 2:** Patient demographic characteristics.

	AMD (*n* = 273)	Control (*n* = 257)	*p*-value
Sex (M/F)	130/143	105/152	0.38
Age (yr)	67.3±7.9	67.6±8.5	0.24
Body Mass Index (kg/m^2^)	24.04±3.15	24.20±3.27	0.64

We attempted to predict the integer value of CN (PCN) at each region and the results were summarized in Table 3. All candidate regions except *ARMS2/HTRA1* showed either CN gain or loss. CN gains in *VEGFA* and *VLDLR* genes had been reported in the previous study on 30 Asian samples. However, CN losses in *VEGFA* and *VLDLR* genes, which were detected in 3 and 29 subjects respectively, were novel findings in the Asian population.

**Table 3:** Association between AMD cases and controls.

		AMD	Control	*p*-value
*VEGFA*				
	Gain	1	0	0.33
	Normal	272	254	
	Loss	0	3	0.074
*ARMS2/HTRA1*				
	Gain	0	0	n/a
	Normal	273	257	
	Loss	0	0	n/a
*CFH*				
	Gain	0	0	n/a
	Normal	265	253	
	Loss	1	0	0.33
*VLDLR*				
	Gain	63	82	0.025
	Normal	184	142	
	Loss	20	29	0.12

**Figure 1:**
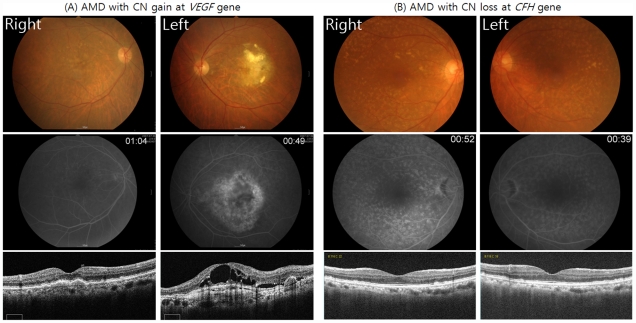
Fundus photographs in patients with copy number (CN) gain or loss. A 78-year-old man with CN gain at *VEGFA* showed typical features of neovascular AMD; with choroidal neovascularization in the left eye and confluent drusen in the right eye (A). Fluorescein angiography and spectral domain optical coherence tomography (SD-OCT) show choroidal neovascularization with cystoid macular edema at the center of the choroidal neovascularization in the left eye. The patient with CN loss at *CFH* gene was a 66-year-old woman with confluent soft drusen in both eyes (B). Flourescein angiography shows multiple hyper-fluorescent lesions at macula of both eyes and SD-OCT shows clumps in the neurosensory retina and focal elevations of retinal pigment epithelial layer.

In AMD patients, CN loss of *VEGFA* gene was not detected and one CN gain was found. The patient with CN gain of *VEGFA* gene had neovascular AMD in the left eye and intermediate AMD in the right eye (Figure 1A). Three subjects in the control group showed CN losses in *VEGFA*, whereas no CN gain was detected. No AMD patient or control subject presented with CN gain at *CFH*, and only 1 AMD patient presented with CN loss. The patient with CN loss at *CFH* had intermediate AMD with confluent drusen in both eyes (Figure 1B). CN gain and loss in *VLDLR* gene were more common compared to other regions, contributing more than one third of total subjects. The number of CN gain in *VLDLR* gene was much higher in control subjects than in AMD patients (p = 0.025), while CN loss was not significantly different between the patient and control groups.

To overcome false negative calls that could be generated in the process of CN prediction, we also analyzed the mean RCV which was not a whole number. When the mean RCV of each region was analyzed between the patient and control groups, patients with AMD had significantly higher RCV than controls at *VEGFA* and *CFH* (2.03 *vs.* 1.92 for *VEGFA*, *p*<0.01, 2.01 *vs.* 1.97 for *CFH*, *p*<0.01), and lower RCV at *ARMS2/HTRA1* (1.97 *vs.* 2.01, *p*<0.01, Table 4).

**Table 4:** Mean copy number in AMD cases and controls.

Genes	AMD	Control	*p*-value
*VEGFA*	2.03	1.92	<0.01
*ARMS2/HTRA1*	1.97	2.01	<0.01
*CFH*	2.01	1.97	<0.01
*VLDLR*	2.14	2.22	0.176

## Discussion

Recent studies have suggested that CNVs play important roles in the development of inheritable diseases [14]. Although less abundant than SNP, CNV seems to account for more nucleotide variation due to its sheer size [15]. By spanning thousands of bases, CNVs often encompass functional DNA sequences. A recent comparison of the relative impact of SNP and CNV on gene expression identified that a substantial proportion (∼18%) of gene expression variability might be attributable to known CNVs [15].

In this study, we found three out of four candidate regions showed CNVs in AMD patients or control subjects. CNV frequencies in some regions were found to differ from those of our previous screening study. For instance, we previously found loss of *CFH* in 5 out of 30 individuals. In this study, however, none of the AMD patients and control subjects demonstrated CN loss at this site. Ethnic differences may be responsible for this discrepancy. In case of *CFH* loss, every CNV loss was discovered in the Chinese and Japanese, yet not in Koreans. The detection of CNV could be platform-dependent as well. A previous study discovered CNVs using array CGH, whereas this study used TaqMan probes based on real-time PCR. Furthermore, we used the CopyCaller program and maximum-likelihood algorithm to determine CN or predicted CN, and set the most frequent CN at 2. However, if there is a region with more than 2 copies in the actual human genome, its CN determination would have a number of false results. Regions with high frequencies of CNV, such as *VLDLR* in our study, could be suspected as multi-copy loci.

Considering factors mentioned above, the issue of false CNV may be the first hurdle to overcome for this kind of association study. In addition to the duplication of experiments, we repeated experiments for samples with CN gain and loss to avoid false positive CNV calls, and confirmed whether they were consistent with previous experiments. CN gain or loss of *VLDLR*, however, was excluded from these re-experiments, since this region appeared to be highly CNV-prone in both patient and control groups. CN gains and losses of other three regions shown in Table 4 were all confirmed by two separate experiments. Besides, all the plates were designed as almost an equal number of patient and control samples since false calls might occur randomly in each plate.

PCN would be the most apparent and decisive value showing the CNV status of each individual if RCV is converted well into PCN without error. However, we found several cases showing borderline RCVs, which make it difficult to determine whether they have two copies or not. To overcome these kinds of false negative or positive calls, we also compared the mean RCV between patient and control groups and showed the possible association of candidate genes with the AMD development.

Vascular endothelial growth factor (VEGF) is a major molecular mediator of neovascularization. Intraocular VEGF expression was found to be increased in neovascular AMD patients, which led to the development of VEGF inhibition therapies with anti-VEGF antibodies for neovascular AMD [16]. However, studies analyzing SNP did not show consistent results [9], [17]. In this study, CN loss in *VEGFA* was discovered in 3 control subjects, which had not been reported in the Asian or Caucasian populations before. Although our study analyzed a relatively large number of AMD patients and controls, further investigations should be conducted in other ethnic groups to confirm the possible effects of CNV on *VEGFA* gene in AMD development. In addition, because the CNV in *VEGFA* gene appeared to be rare in the Korean population, larger sample sizes are required for further studies.

The *ARMS2/HTRA1* gene encodes a member of a family of serine proteases expressed in both mouse and human retinas, and its expression in human fibroblasts increases with aging [18]. Over-expression of *ARMS2/HTRA1* alters the integrity of the Bruch's membrane, favoring the invasion of choroid capillaries across the extracellular matrix, as occurs in wet AMD [19]. In this study, on the contrary, the mean RCV at *ARMS2/HTRA1* was lower in AMD patients compared to control subjects. Since there was no CN gain or loss predicted, studies to elucidate the genuine CNV status of *ARMS2/HTRA1* in AMD patients, and its possible effect on *ARMS2/HTRA1* expression would be needed.

Genes encoding complement factors have been identified as AMD susceptibility loci with convincing statistical evidence. They include complement factor H gene (*CFH*) on chromosome 1q32 [20], complement component 3 gene on 19p13 [21], and 2 neighboring genes on 6p21 (complement factor B and complement component 2) [17], [19]. This study discovered CN loss of *CFH* gene in one AMD patient but it could not find any association of *CFH* variation with AMD. Other complement factor genes and complement factor related regions may find significant genetic variations in complement factor related genes in AMD patients.

Very low density lipoprotein receptor (VLDLR) is involved in lipid transportation and chronic inflammation through the Wnt pathway [22], [23]. *VLDLR* gene knockout (*VLDLR*−/−) mice were shown to develop sub-retinal neovascularization [24], [25] and the features of neovascularization resemble those of retinal angiomatous proliferation in human — a subtype of AMD [17]. In an association study, variations (rs10967213, rs2290465) of *VLDLR* showed associations with AMD prevalence [26], [27]. In this study, the CN gain or loss of *VLDLR* was very common in control subjects and in AMD patients. It may be due to its regional complexity in the human genome, and therefore this region should be interpreted with caution.

In this study, we selected target CNVs out of Asian-specific CNVs discovered in our previous study. Consequently, several genes which have been thought to play important roles in AMD pathogenesis and widely studied in other ethnic groups, such as *C2/BF*, *TIMP*, *CFHR1* and *CFHR3*, were not included in our target regions.

In conclusion, the outcome of this study suggests that some CNVs might be associated with AMD development, although the actual action of these CNVs *in vivo* remains unelucidated. Further CNV-oriented research is required to improve our understanding of the mechanism that underlies AMD.

## Supporting Information

Table S1 General information of TaqMan Copy Number Assays used for this study.
(DOC)
