# Supplementary material for: Copy Number Variation of Age-Related Macular Degeneration Relevant Genes in the Korean Population
Source: PLoS One. 2012 Feb 15;7(2):e31243. doi: 10.1371/journal.pone.0031243 (PMC3280288; doi:10.1371/journal.pone.0031243)
Supplement: Table S1 — General information of TaqMan Copy Number Assays used for this study. (DOC) [file pone.0031243.s001.doc]

Table S1. General information of TaqMan Copy Number Assays used for this study

| **Gene Symbol** | **Assay ID** | **Context Sequence** | **Cytogenetic Band** | **NCBI Assembly Build Number** | **Location on NCBI Assembly** |
| --- | --- | --- | --- | --- | --- |
| *HTRA1* | Hs05220034_cn | CTGGTCCCTCGCCACAGGATAATTA | 10q26.13b | 36 | 124218533 |
| *VEGFA* | Hs00660149_cn | TTCTCGCTTCGGAGGAGCCGTGGTC | 6p21.1c | 36 | 43846617 |
| *VLDLR* | Hs06893853_cn | GCTCAAAGGAGCAGCGCGAGAGCGA | 9p24.2b | 36 | 2613145 |
| *CFH* | Hs04191996_cn | GTAAACAGGGACTCTAGAAATTCAT | 1q31.3c | 36 | 194976633 |
